# Supplementary material for: Fecal Microbiota Transplant in Two Ulcerative Colitis Pediatric Cases: Gut Microbiota and Clinical Course Correlations
Source: Microorganisms. 2020 Sep 27;8(10):1486. doi: 10.3390/microorganisms8101486 (PMC7599854; doi:10.3390/microorganisms8101486)
Supplement: Supplementary file 1 [file microorganisms-08-01486-s001.pdf]

Supplementary information.

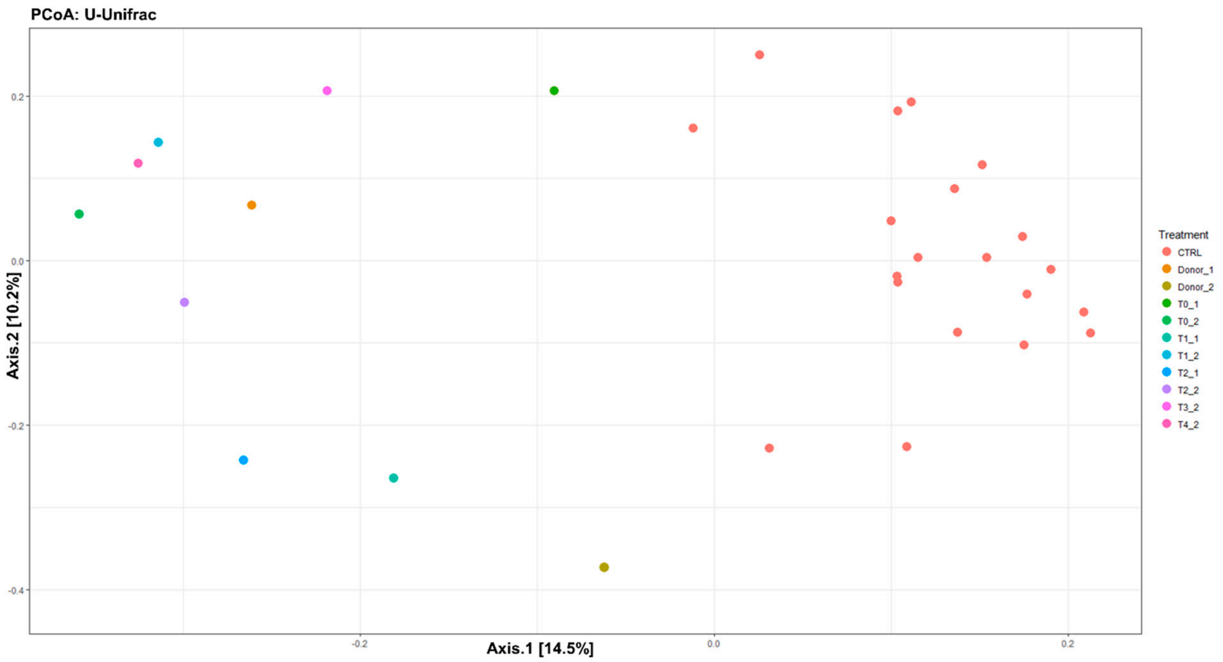

Supplementary figure S1: Unweighted UniFrac analyses on the whole dataset. Samples from recipients, donors and controls were analyzed through Unweighted UniFrac analysis. Two main clusters are evident: one composed by all recipients' samples and one by donors and control samples.

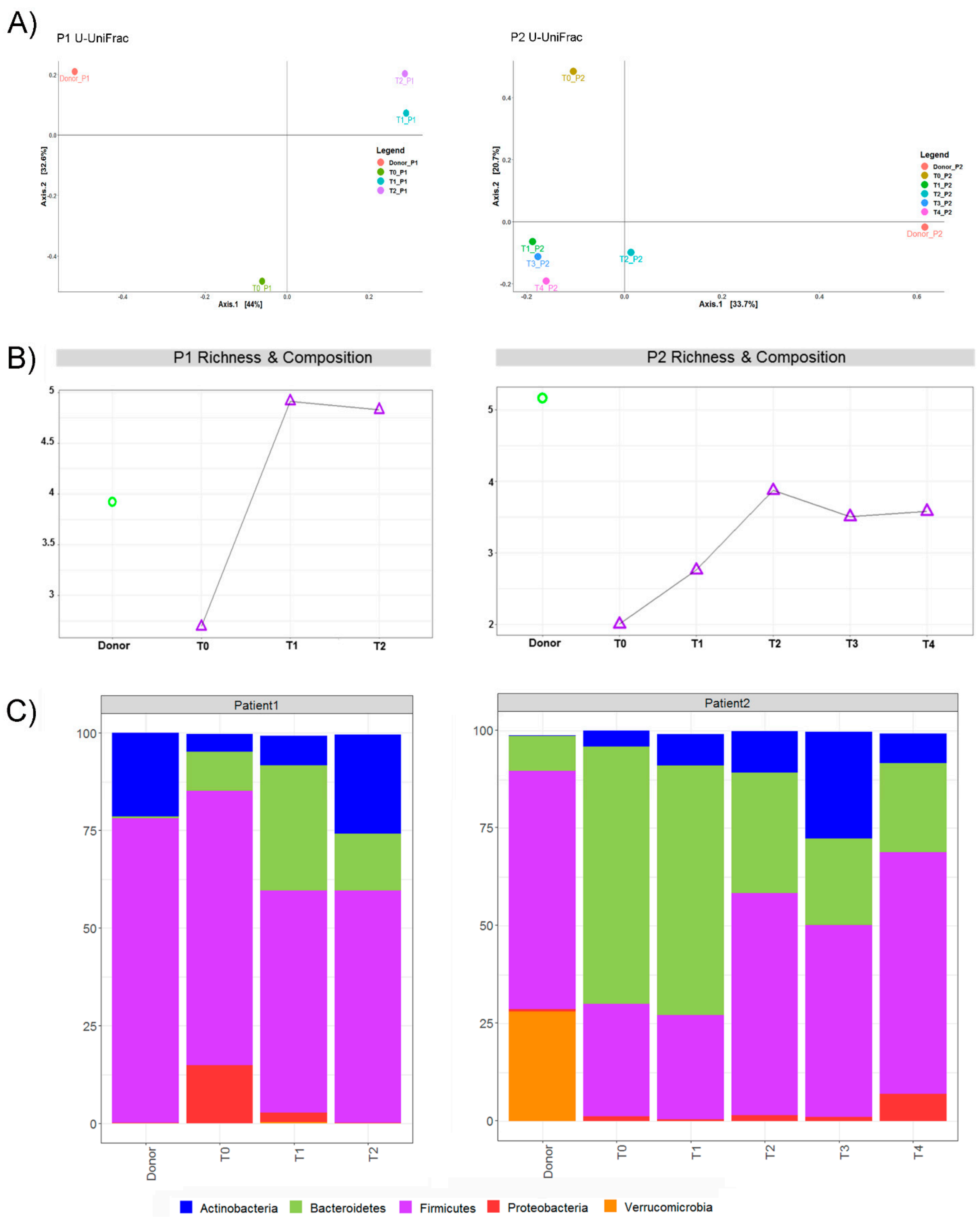

Supplementary figure S2: Microbiota profile variations during time. A) Unweighted UniFrac analyses for P1 and P2 with their respective donor (red dot) samples. In Panel B, Shannon index from α-diversity analysis is reported for both patients (violet triangles) and donors (green circles). Finally, in Panel C, bar plots show microbiota variation at phylum level for each sample.

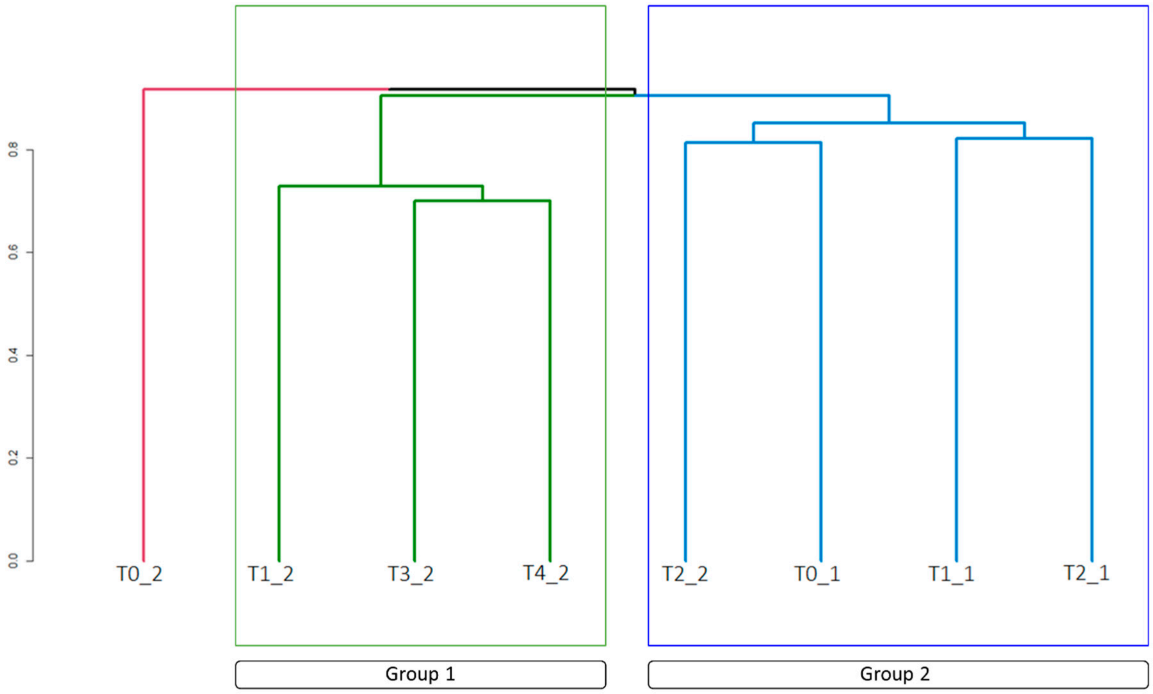

Supplementary figure S3: Hierarchical cluster analysis based on Jaccard Distance among patients' samples. This analysis depicts two main clusters based on bacterial ecology: group 1 (green box) composed by almost all samples of P2 and group 2 (blue box) by all P1 samples and T_2 sample from P2.

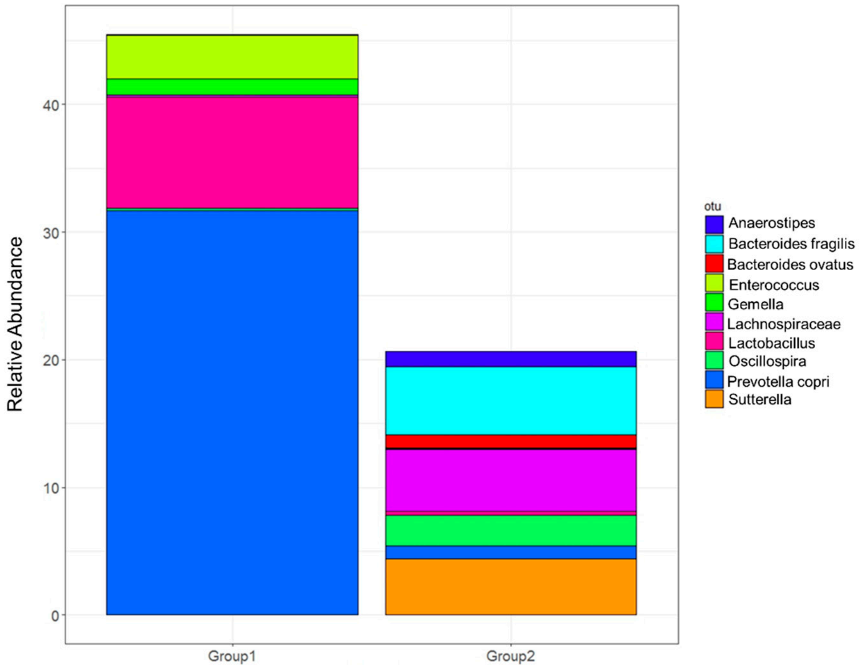

Supplementary figure S4: Mean relative abundance of significant species (DESeq2 FDR-p<0.05) in groups 1 and 2 as detected by hierarchical cluster analysis. Only bacterial genera with a relative abundance greater than 1% are displayed.
